# Supplementary material for: Genome-wide correlation analysis to identify amplitude regulators of circadian transcriptome output
Source: Sci Rep. 2020 Dec 14;10:21839. doi: 10.1038/s41598-020-78851-9 (PMC7736363; doi:10.1038/s41598-020-78851-9)

Supplementary Information

Genome-wide correlation analysis reveals *Rorc* as potential amplitude regulator of circadian transcriptome output

Evan S. Littleton, Maddison L. Childress, Michaela L. Gosting, Ayana N. Jackson, Shihoko Kojima*

Department of Biological Sciences, Fralin Life Sciences Institute, Virginia Tech, Blacksburg, VA, USA

Supplemental Figure Legends

Figure S1. Characteristics of mouse circadian transcriptome (RNA-seq) in various mouse tissues. (A) Percentage of cycling genes in each tissue from highest (left) to lowest (right) % cycling. Rhythmicity of a gene was defined as Benjamini-Hochberg q values < 0.05 by MetaCycle. (B) Distribution of Benjamini-Hochberg q values of all expressed genes in each tissue. (C) Numbers of genes expressed in each tissue. (D) Average microarray signals per gene for all probesets. (E) Distribution of relative amplitude of cycling genes in each tissue calculated by MetaCycle. (F) Distribution of the amplitude of cycling genes in each tissue calculated by MetaCycle. (D-F) The central line represents the median, and each box represents the 25th and 75th percentiles, respectively. The notch represents a 95% confidence interval around the median. Numbers of expressed genes or rhythmic genes in each tissue can be found in the Supplementary Data Sheet 2. Each color corresponds to a tissue; liver (purple), kidney (light purple), lung (blue), brown adipose (BAT) (light blue), heart (green), adrenal (light green), aorta (yellow), cerebellum (gold), hypothalamus (orange), muscle (coral), and brainstem (dark red).

Figure S2. Correlation between the percentage of cycling genes and the expression patterns of core clock genes in each tissue in RNA-seq data. (A) Colors of each tissue correspond to Fig. 1; liver (purple), kidney (light purple), lung (blue), brown adipose (light blue), heart (green), adrenal (light green), aorta (yellow), cerebellum (gold), hypothalamus (orange), muscle (coral), white adipose (red), brainstem (dark red). (B) Single value decomposition (SVD) of tissues (left) or core clock genes (right) in RNA-seq. Eigentissues and eigengenes 1 and 2 were projected onto clock gene expression values, *Rorc* and *Per2AS* indicated in red and grey, respectively.

Figure S3: Genome-wide correlation analysis using baboon datasets (Mure et al., Science 2018). (A-C) Correlation between the mean level of *Rorc* and the percentage of rhythmic genes in 13 baboon tissues using MetaCycle (A) or JTK-CYCLE (B), or in 12 mouse tissues determined by JTK-CYCLE (C). (D) UCSC genome bowser view of *PER2* (red) and *PER2AS* (black) expressions in baboon from lung (top) and prostate (bottom).

Supplemental Figure 1. Littleton et al

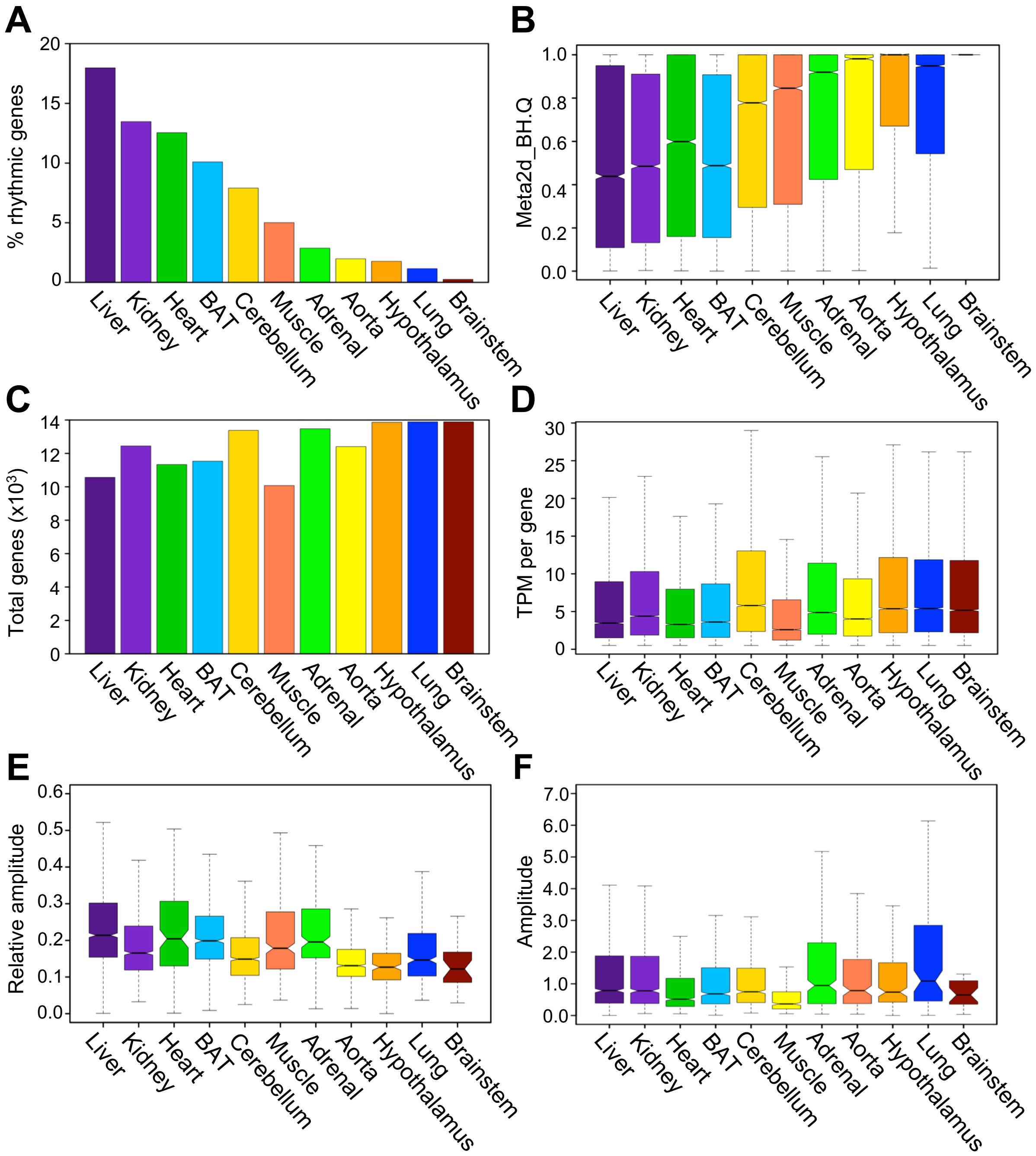

Supplemental Figure 2. Littleton et al

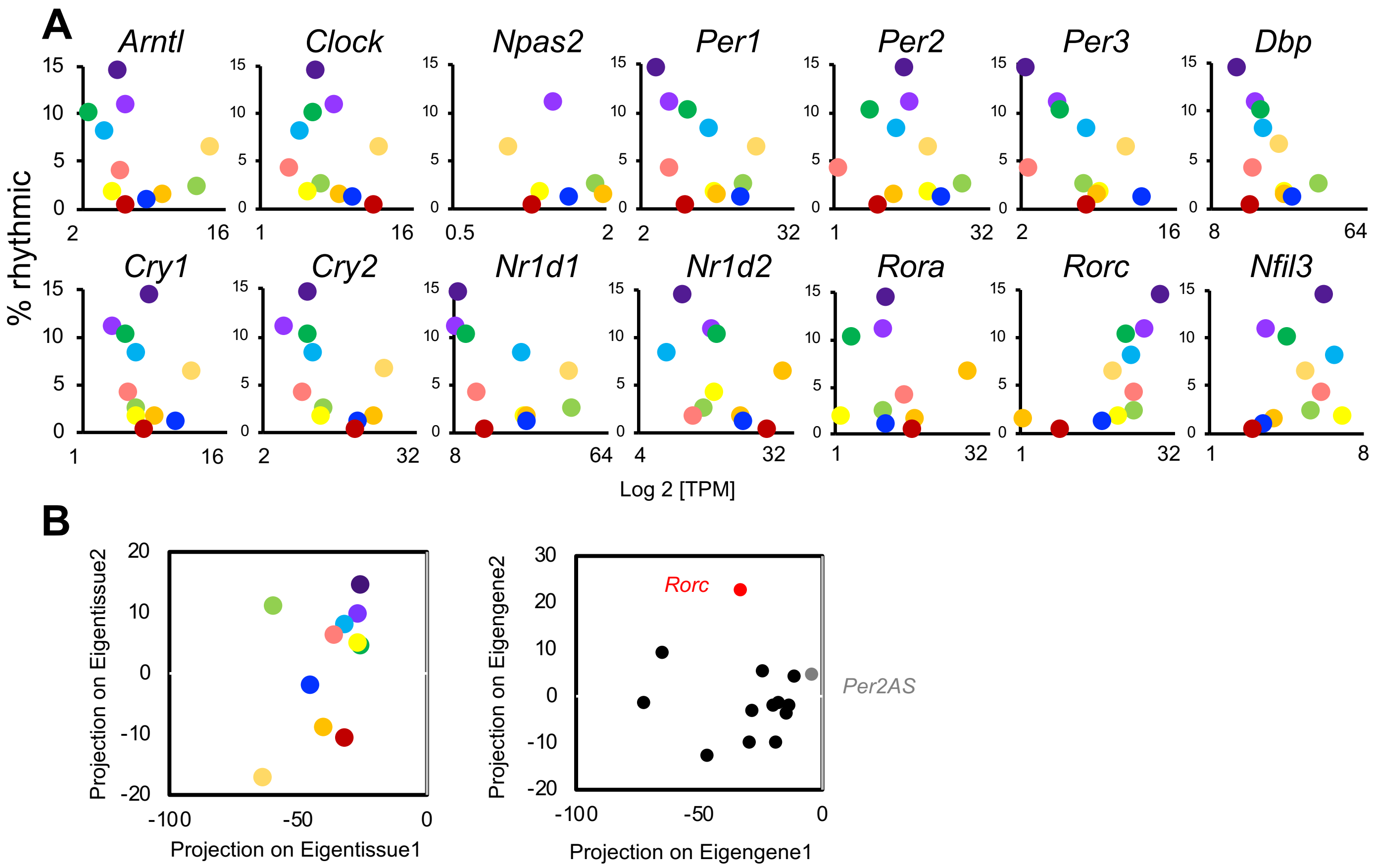

Supplemental Figure 3. Littleton et al

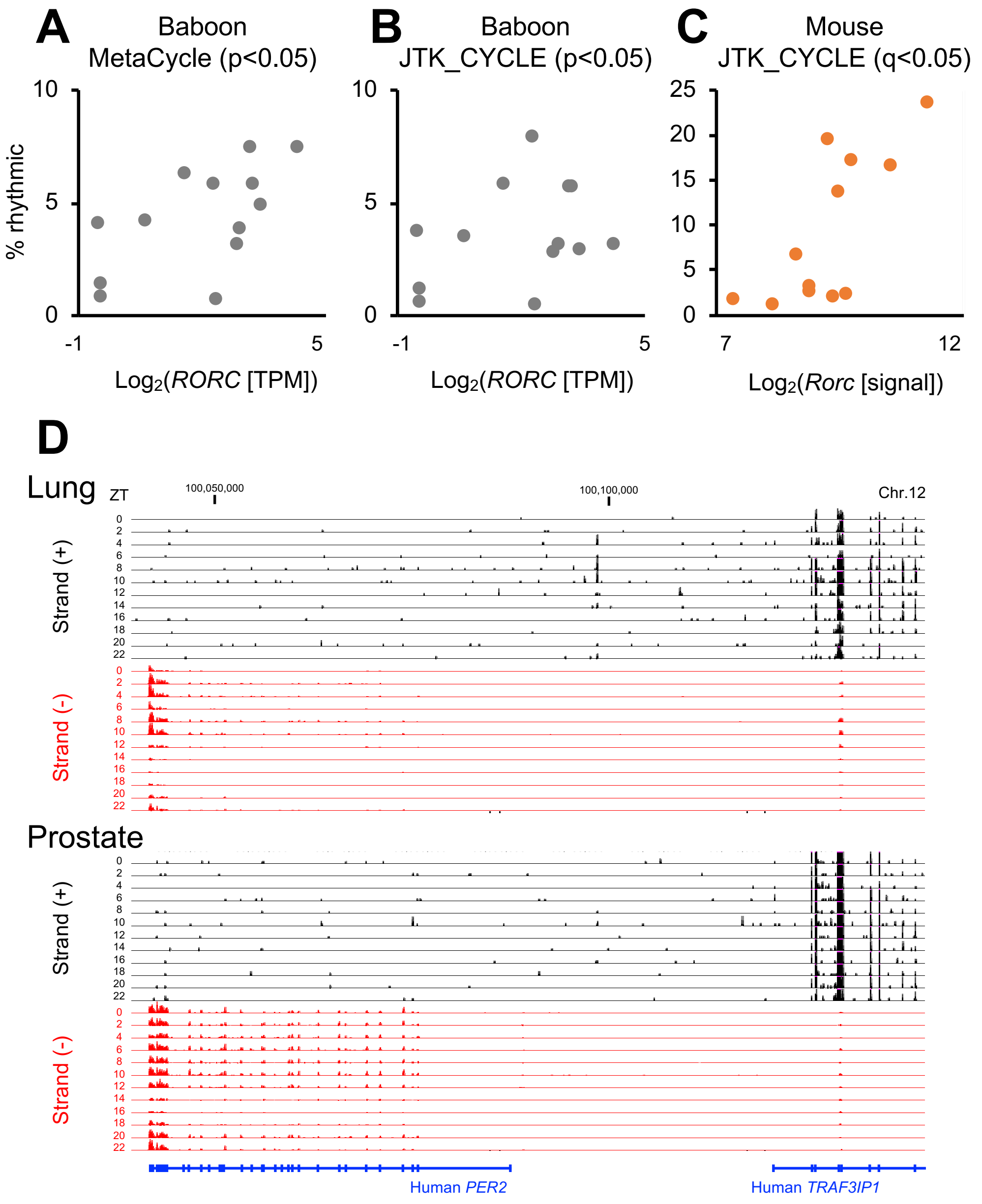

Supplemental Figure 2. Littleton and Kojima

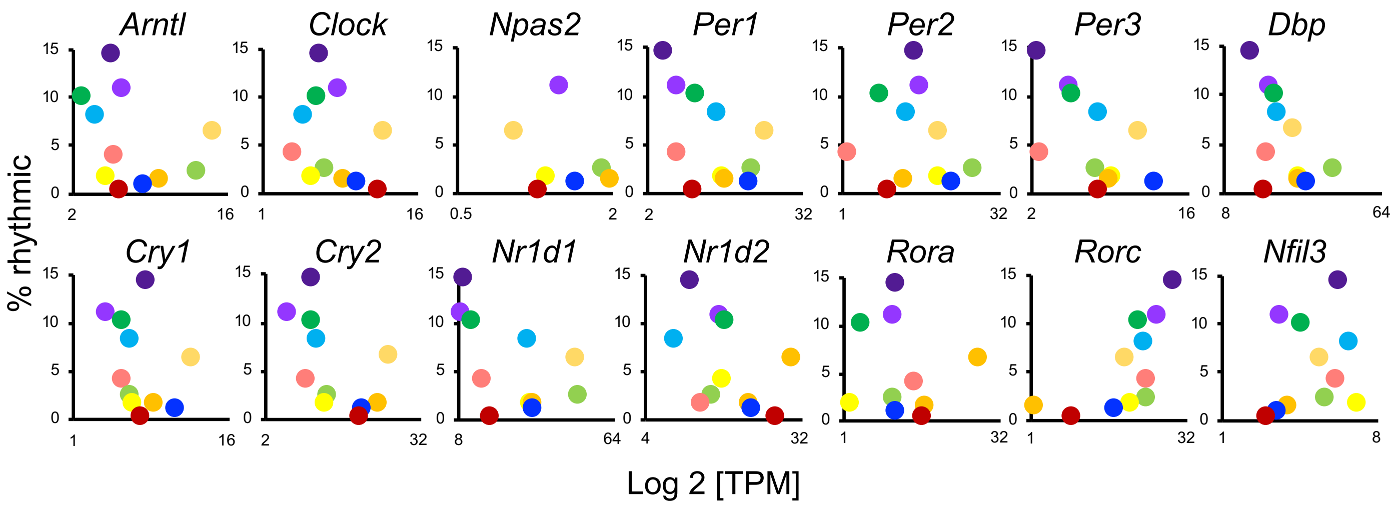

Figure 4. Littleton and Kojima

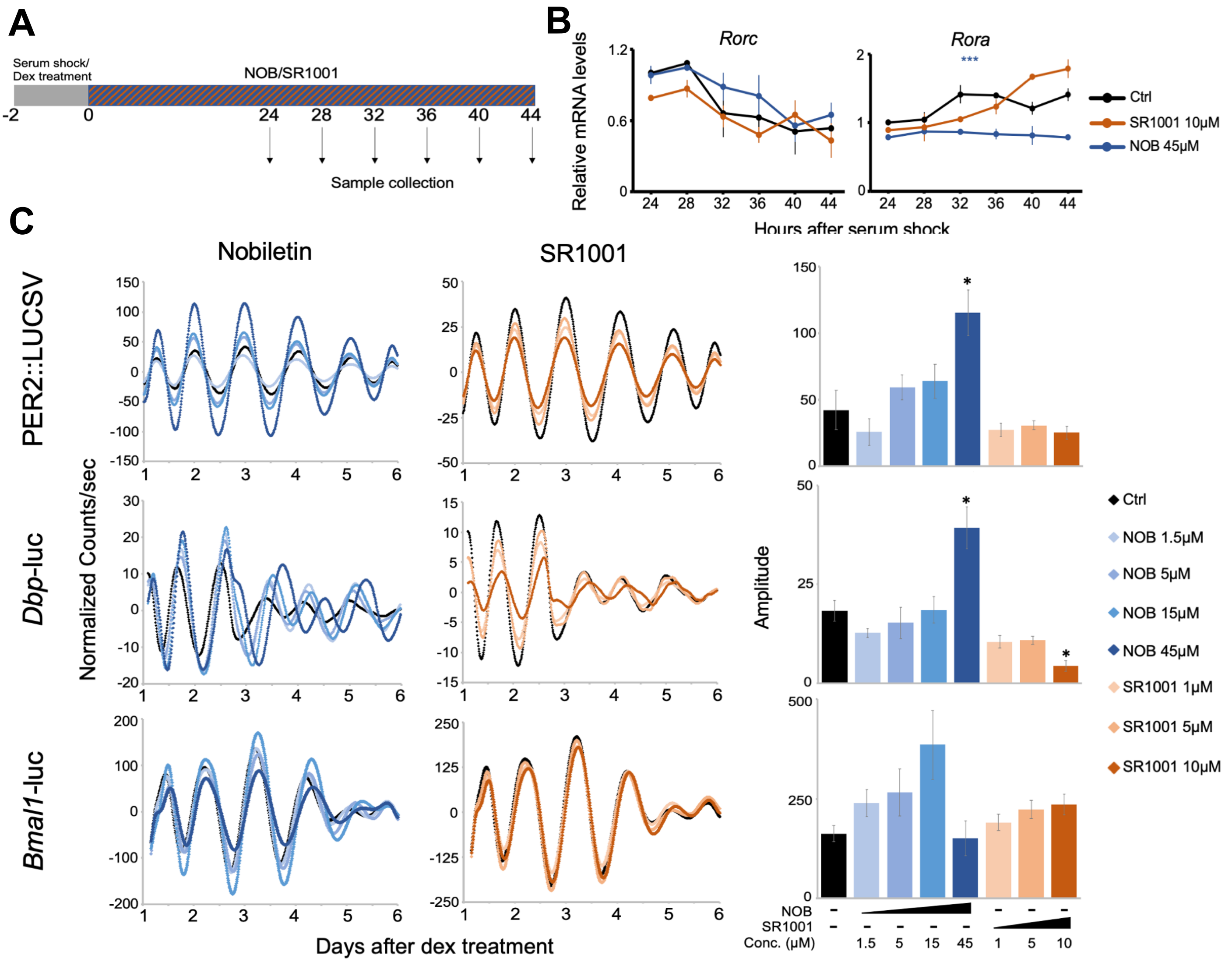

Supplement: Supplementary file 1 — Supplementary Figures. [file 41598_2020_78851_MOESM1_ESM.pdf]
